# Merkel cell carcinoma disguised as a ganglion cyst in a pianist: Oncologic management and functional reconstruction

**DOI:** 10.1016/j.jdcr.2024.09.014

**Published:** 2024-10-01

**Authors:** Anna Carmichael, Taylor Martin, Thomas Soike, Carlos Floresguerra, Jeremy Powers

**Affiliations:** Department of Surgery, Quillen College of Medicine, East Tennessee State University, Johnson City, Tennessee

**Keywords:** atypical presentation, differential diagnosis, ganglion cyst, Merkel cell carcinoma, neuroendocrine tumor

## Introduction

Merkel cell carcinoma (MCC) is a rare and aggressive type of skin cancer arising from Merkel cells, which serve as mechanoreceptors along the dermal-epidermal junction. Compared to melanoma, MCC has a worse 5-year prognosis and is more likely to metastasize. MCC arises in areas of sun distribution on the skin, particularly in elderly and immunosuppressed populations.^1^

It is proposed that the initial pathogenesis of MCC is ultraviolet radiation exposure and the second is Merkel cell polyomavirus.^1^ Risk factors for MCC include Caucasian ethnicity, male sex, age greater than 70, immunocompromised states including human immunodeficiency virus and solid organ transplant, and lymphoproliferative disorders including chronic lymphocytic leukemia (CLL) and small lymphocytic lymphoma.^1^^,^^2^

We present a case of MCC with the initial clinical appearance of a dorsal wrist ganglion cyst.^3^ The authors additionally review the literature for other clinically atypical presentations of MCC.

## Case report

**Fig 1:**
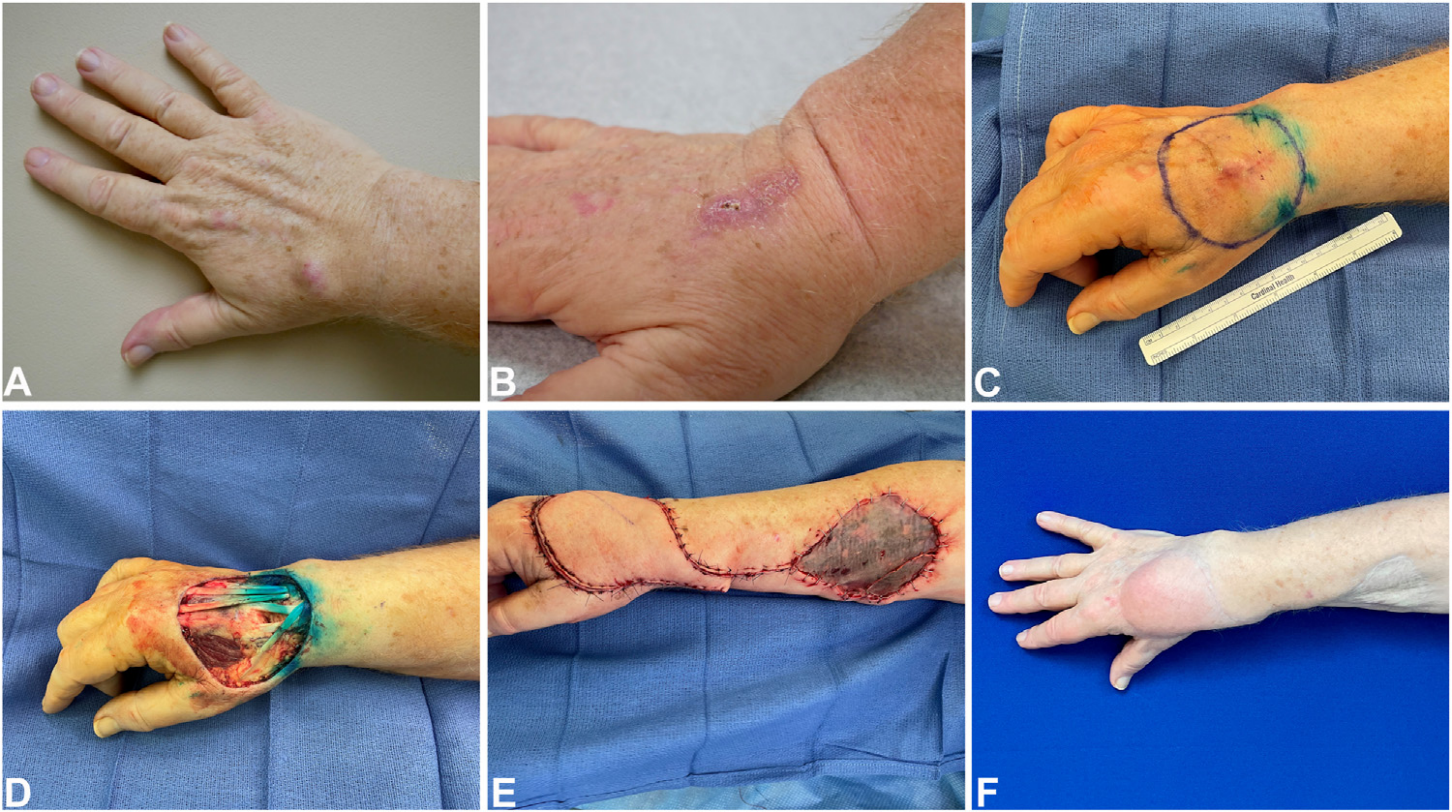
**A,** Initial clinic visit presentation of subcutaneous nodule on right dorsal wrist, (**B**) clinical presentation 3 weeks after excisional biopsy, (**C**) wide local excision markins and blue dye from sentinel lymph node injection (**D**) wide local excision defect with exposed tendons, (**E**) immediately postop radial forearm flap and full thickness skin graft, and (**F**) 15 months postop plastic surgery clinic visit.

A 72-year-old male with past medical history of diabetes, hypertension, obesity, CLL and prostate cancer in remission was referred to the plastic surgery clinic in September 2021 for a presumed ganglion cyst on the dorsal aspect of his right wrist, which had been present for a few months and caused pain while playing the piano (Fig 1). Surgery was initially delayed due to elective surgery restrictions during the COVID-19 pandemic. Due to increased lesion size and pain over the next few weeks, surgery was scheduled more urgently.

**Fig 2:**
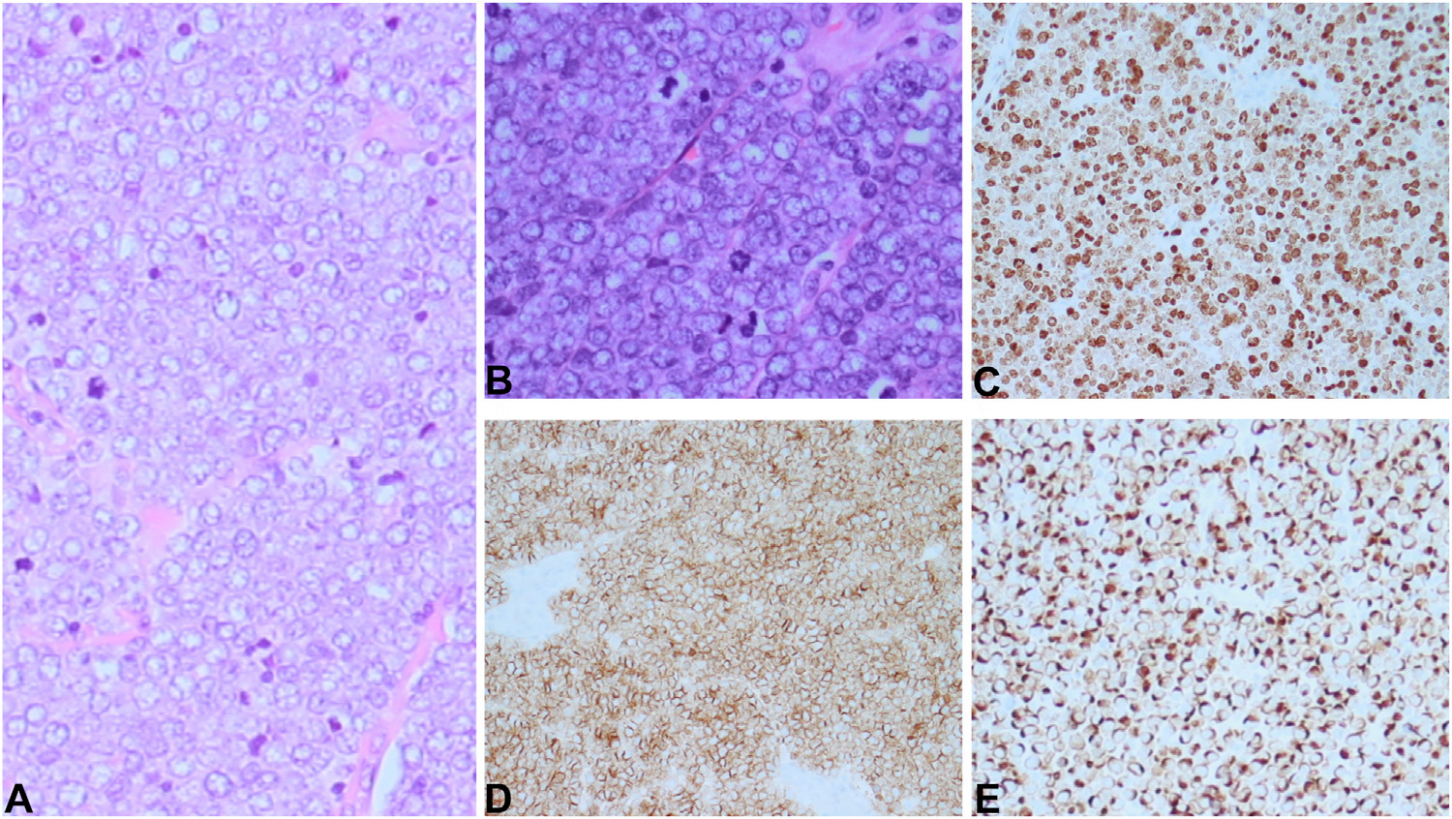
**A,** Hematoxylin and eosin stain 200× magnification (**B**) hematoxylin and eosin stain 400× magnification revealing numerous mitotic figures, nuclear features show stippled chromatin (**C**) Ki-67 stain showing 90% proliferation rate (**D**) CD 56 stain positive (**E**) cytokeratin 20 stain showing perinuclear dot-like positivity.

A solid fibrous mass was found after incision through the skin, and the procedure converted to an incisional biopsy. Pathology results showed sheets and nests of malignant epithelioid neoplasm composed of cells with monotonous small and intermediate-sized round nuclei with clear and scant cytoplasm and visible mitoses. The neoplastic cells stained positive for keratin AE1/AE3, CAM5.2, CK20 (perinuclear dot-like positivity), CD56, synaptophysin, chromogranin, and BCL2. Ki-67 showed an increased proliferation rate of 90% to 95%. These histopathologic features were consistent with MCC (Fig 2). Oncology was consulted, and positron emission tomography showed no evidence of metastatic disease. Per National Comprehensive Cancer Network Guidelines,^2^ the patient was treated as stage N0 and scheduled for wide excision with 2 cm margins and sentinel lymph node biopsy (Fig 1). A skin substitute was placed to protect the exposed extensor tendons while awaiting margin assessment. Margins were reported as clear, and sentinel nodes were negative.

After clear margins were established, a vascularized flap was chosen for reconstruction due to planned adjuvant radiation therapy and need for optimal function over the exposed tendons. A reverse radial forearm flap to the dorsal wrist defect was performed and a split thickness skin graft from the thigh was applied to the volar proximal forearm donor site (Fig 1). After a short period of immobilization for graft healing, he was referred to hand therapy for wrist and finger range of motion, with the goal to preserve his ability to play the piano.

Adjuvant radiation therapy was administered to the right hand and wrist. At 3-month follow-up, the patient was healing well without signs of recurrence and observation was pursued per the National Comprehensive Cancer Network guidelines.

At a routine plastic surgery follow-up 4 months later, a mass was palpated in the right axilla. An urgent core needle biopsy was performed and confirmed metastatic MCC. Positron emission tomography scan at that time showed right lateral chest/axillary radiolabel uptake. He underwent complete axillary lymph node dissection 2 weeks later with adjuvant radiation therapy to his right axilla per National Comprehensive Cancer Network guidelines for clinical node positive disease.^2^ Two of six dissected nodes were found to be positive for MCC. Given the patient’s high-risk disease, early recurrent disease, and risk factors including history of CLL, the patient was started on pembrolizumab. He completed one year of therapy with 9 cycles. Routine positron emission tomography scans at 2 year follow up were negative for metastasis. The patient has followed-up regularly with oncology and plastic surgery without evidence of disease recurrence. Despite major soft tissue surgery on the hand and wrist as well as radiation therapy, he achieved satisfactory functional outcome and has maintained the ability to play the piano.

## Discussion

MCC is exceptionally difficult to diagnose given its clinical and histologic similarities to many other dermatologic conditions. MCC typically presents as a firm, violaceous nodule. These nodules can demonstrate hyperkeratosis, ulceration, telangiectasia, and/or shiny surfaces. The differential diagnosis for MCC includes basal cell carcinoma, amelanotic melanoma, lipoma, epidermal inclusion cyst, lymphoma, and atypical fibroxanthoma.^1^

A review of the literature reveals only one other reported case of MCC presenting as a ganglion cyst with no apparent epidermal changes.^3^ This might represent an atypical clinical presentation of MCC. Other atypical clinical presentations of MCC found in the literature include atheroma, gluteal ulcer, arthropod bite reaction, and ptosis and swelling of the upper eyelid without discoloration of the skin but with a multinodular mass beneath the conjunctival surface.^4^, ^5^, ^6^, ^7^

According to previous studies, the incidence of MCC increases with age, and is nearly 3 times more prevalent in males than in females.^8^ This appears to be consistent with the 2 cases of clinically atypical MCC presenting as ganglion cysts. In both cases, the patients were 72-year-old males.^3^ In the other clinically atypical cases of MCC, the age range at presentation was 55-85 years of age with an even number of presentations between males and females.^4^, ^5^, ^6^, ^7^ MCC has also been shown to be associated with lymphoproliferative disorders such as CLL,^8^ which was present in our patient but not reported in the case reported by Rothrock et al.

While the number of clinically atypical cases of MCC reported in the literature is limited, it is important that MCC be considered in the differential diagnosis of potentially benign-appearing lesions. This is crucial when presenting in elderly patients with comorbidities predisposing to immunosuppression. For such patients, the threshold for excisional biopsy should be lowered. Furthermore, close clinical follow-up with surveillance physical exams for lymphadenopathy is essential to detect recurrence or metastasis.

## Conflicts of interest

None disclosed.
